# Association Between Self‐Reported Oral Health and Dental Fear Among Illicit Drug Users in Northern Finland

**DOI:** 10.1002/cre2.70055

**Published:** 2024-12-17

**Authors:** Raija Vainionpää, Antti Tiisanoja, Outi Kokkola, Pirkko Riipinen, Vuokko Anttonen

**Affiliations:** ^1^ Faculty of Medicine, Research Unit of Population Health University of Oulu Oulu Finland; ^2^ Social and Health Services Oulu Finland; ^3^ Medical Research Center and University Hospital of Oulu University of Oulu Oulu Finland; ^4^ Department of Psychiatry, Institute of Clinical Medicine University of Oulu Oulu Finland

**Keywords:** dental fear, illicit drug use, MDAS (Modified Dental Anxiety Scale), oral health

## Abstract

**Objectives:**

About 10% of adults in northern Finland have severe dental fear, but there is a lack of detailed knowledge about dental fear among illicit drug users. The aim of the study was to evaluate the prevalence of dental fear and its association with self‐reported oral health and health behavior as well as background factors of the customers of substance abuse services living in the region of Oulu, Northern Ostrobothnia, Finland.

**Materials and Methods:**

One hundred seventeen volunteers, either active or former illicit drug users, were interviewed face‐to‐face about their background factors, health and health behaviors, and use of illicit drugs. The Modified Dental Anxiety Scale (MDAS) was used to assess a situation‐specific level of dental anxiety. For analyses, Pearson's *χ* ^2^ test, Fisher's exact test, ANOVA, and logistic regression analysis were performed.

**Results:**

Three out of four participants had at least moderate dental fear (MDAS ≥ 10) and 24% had severe dental fear (MDAS ≥ 19), with the average MDAS being 14.0 (SD 5.7). Participants in drug rehabilitation reported significantly more dental fear than the rest of the participants. Severe dental fear was associated (adjusted) with previous painful experiences OR 10.8 (2.3–52.0) and poor behavior by dental personnel OR 4.1 (1.2–13.9).

**Conclusions:**

Dental fear is common among illicit drug users, and it is, particularly, associated with previous painful experiences and poor behavior by dental personnel.

## 1 Introduction

Patients with dental fear and those with substance abuse problems can be challenging for dental professionals. Both groups of patients use oral healthcare services irregularly and often only on an as‐needed basis (e.g., when they have problems or pain); they cancel or miss appointments, or avoid seeking dental care completely (Armfield, Spencer, and Stewart 2006; Armfield 2013; Silveira et al. 2021). Furthermore, treating these patients often requires both long appointments and skills from dental professionals due to their general and psychological health problems (Talo Yildirim et al. 2017; Hakeberg and Wide 2019).

Dental fear is a multifactorial phenomenon and is common among the adult population worldwide (Liinavuori et al. 2016; Svensson, Hakeberg, and Boman 2016; Silveira et al. 2021). Interestingly, recent studies have anticipated a decline in the prevalence of self‐reported dental fear (Silveira et al. 2021; Liinavuori et al. 2019). Despite this, the prevalence of dental fear still varies from less than 5% to 50% depending on the study population and cultural, social, and economic differences, as well as the instruments used to measure dental fear (Armfield, Spencer, and Stewart 2006; Cianetti et al. 2017; Silveira et al. 2021). According to a recent meta‐analysis, the prevalence of dental fear in the adult population worldwide is 15%, with the prevalence of severe dental fear being 12% and that of phobia level being 3% (Silveira et al. 2021). In Finland, one in three adults (≥ 30 years) report moderate or mild dental fear and 10% severe dental fear (Pohjola et al. 2008). Among Finns, women report dental fear more often than men and the prevalence of dental fear is the highest in the age group of 30–40 years old (Liinavuori et al. 2016).

The use of illicit drugs is increasing worldwide, and drug use has become a permanent phenomenon (United Nations Office on Drugs and Crime 2023). According to the UN estimate for the year 2021, about 6% of people (aged 15–64 years) have used a drug within the past 12 months (United Nations Office on Drugs and Crime 2023). The use of drugs has also been increasing in Finland and it is estimated that almost a third (29%) of adult Finns have tried or used some kind of drug at some point in their lives. Men report more drug experiences than women (37% vs. 21%). Cannabis has been the most commonly tried or used illicit drug while the proportions of other drugs have been significantly smaller compared to cannabis (Karjalainen, Hakkarainen, and Salasuo 2023).

The literature on dental fear among illicit drug users is scarce. According to Scheutz (1986), dental fear and anxiety were more common among Danish drug abusers (*n* = 41) than in a reference group of the same age (*n* = 350). To advance scientific evidence on the topic, this study evaluated dental fear and its association with self‐reported dental health, dental attendance, and health behaviors, as well as background factors of customers of substance abuse services living in the region of Oulu, Northern Ostrobothnia, Finland.

## 2 Materials and Methods

### 2.1 Participants

**Figure 1:**
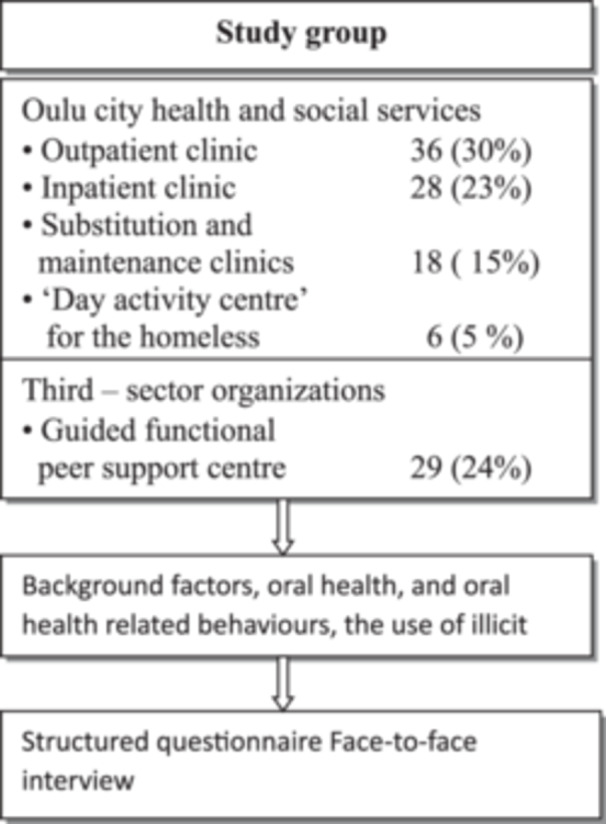
Distribution of participants [*n* (%)] and examination protocol in northern Finnish illicit drug users.

Adult (≥ 18 years) illicit drug users living in the Oulu region of Finland participated in this cross‐sectional field study: 77 men (66%) and 40 women (34%). The mean age of the participants was 33 years (SD 8.0). The participants included both active and former illicit drug users, as well as individuals undergoing pharmaceutical substitution, maintenance therapy, or medically assisted drug rehabilitation. The study population and protocol are described in Figure 1.

A power calculation was performed to determine the size of the study population. A confidence level of *α* = 0.05 and a ratio of 0.1 (10%) were used. The required size of the study population was determined to be 139 participants according to the calculation.

### 2.2 Data Collection

In total, eight different substance abuse service units in the Oulu region were recruited to participate in the study. Six service units were Oulu City Health and Social Services and two units were third‐sector organizations (Figure 1). The staff of the organizations informed their customers about the study and recruited participants for the study. The staff also provided premises for the study. Both written and oral information about the study was given to the participants in advance. Participation was voluntary and informed consent was obtained from all the participants. The data of the study were collected and analyzed without identifying the participants.

Three examiners (A.T., O.K., R.V.) conducted face‐to‐face interviews as well as clinical oral examinations in the service units between October 2020 and May 2022. The field phase was prolonged by the COVID‐19 pandemic. Both a clinical examination and an interview were done at the same time, and the appointment took approximately 75 min.

### 2.3 Interview and Questionnaire

Background factors and health‐related behaviors were surveyed using a structured and validated questionnaire (Anttonen et al. 2012, Vainionpää et al. 2017). The participants' background factors were surveyed as follows: gender (*men/women*), age (*years*), education level (*basic* = 9 years of *compulsory education/second‐degree education = vocational/high school/third degree = university or university of applied sciences*), marital status (*single/in a relationship*), and work–life (*employed/unemployed/retired/student)*. The participants were asked if they had any long‐term (lasting more than 2 months) illness requiring medication (*yes/no*) including mental disorders. The participants were also asked about what illness or diseases they had and if they were using any prescribed drug (open questions). These data were self‐reported; the participants' registered health data were not available to the researchers.

Information on substance use was surveyed with the following questions (Anttonen et al. 2012, Vainionpää et al. 2017): “Do you smoke?” and the alternatives were as follows: *I have never smoked/I smoke daily/almost daily/occasionally/I used to smoke, but I have stopped smoking*. “Do you use snus?”: *I have never tried or used snus/daily or almost daily/occasionally/I used to use, but I have stopped using/I have tried a few times. “*Do you use e‐cigarettes or similar vapers?*”: daily or almost daily/occasionally/I used to use, but I have stopped using/I have tried a few times/I have never used. “*Do you use alcohol?”*: no/less than once a month/approximately once a month/approximately every 2 weeks/approximately once a week/more often than once a week/daily or almost daily*. Alternative answers to illicit drug use were as follows: *I have never used/I have tried once/I have tried two to four times/I have used five times or more/I use regularly*. Additional questions for illicit drug use were as follows: “How long have you used drugs (*years*) and what is the main drug you use?.” “Are you in drug rehabilitation?”: *yes/no*.

Dental fear was assessed with the question (Pohjola et al. 2008): “Are you afraid of dental treatments (*yes/no*)”? The reasons for dental fear were clarified by asking (Vainionpää et al. 2019): “In your own opinion, what are the reasons for your dental fear”: *painful or difficult experiences with previous dental care/poor experiences with dental care of your family or friends/excessive dental treatment need/general health and psychic problems/poor behavior of dental personnel*.

To assess the participants' situation‐specific level of dental fear/anxiety, the Modified Dental Anxiety Scale (MDAS) was used (Humphris, Morrison, and Lindsay 1995). The MDAS is a validated and widely used method for assessing dental fear and anxiety. It consists of a validated and reliable questionnaire with five items that are used to measure imagined reactions to five different dental situations. The five items are as follows: (1) “If you went to your dentist for treatment tomorrow, how would you feel?”; (2) “If you were sitting in the waiting room (waiting for treatment), how would you feel?”; (3) “If you were about to have a tooth drilled, how would you feel?”; (4) “If you were about to have your teeth scaled and polished, how would you feel?”; and (5) “If you were about to receive a local anesthetic injection in your gum above an upper back tooth, how would you feel?.” There are five different response options for all the questions, ranging from 1 (*not anxious at all*) to 5 (*extremely anxious*). The total sum scores of the answers range from 5 to 25 and are usually categorized as follows: 5–9 (*not anxious at all*), 10–18 (*moderately anxious*), and 19–25 (*extremely anxious*) (Yuan et al. 2008).

The participants were asked about their subjective oral health with the following questions: “What is the condition of your teeth now?” with the response alternatives: *good/rather good/average/and rather bad/bad*. In your opinion, do you have (with an answer option of *yes/no*)*: cavities/bleeding of gums when brushing/tooth or teeth needing extractions/pain or other symptoms/healthy dentition that does not need treatment*? “How satisfied you are with your dental appearance?”: *extremely satisfied/satisfied/rather satisfied/unsatisfied/rather unsatisfied/extremely unsatisfied*. Previous dental treatment was assessed with the question: “Have you had teeth repaired with fillings, undergone scaling, received individual dietary counseling, individual oral hygiene counseling, undergone orthodontic treatment, had teeth extracted?” The answer alternatives for all these were *yes/no*. Furthermore, the participants were asked if nitrogen oxide, conscious oral sedation, or general anesthesia had been used previously in dental care due to dental fear *(yes/no)*. The regularity of dental attendance was asked with the questions: “Do you usually go for dental treatment?*”: as needed, for example, due to pain/regularly for checking the dentition/never*. *“*How long has it been since your last dental visit”: *less than 1 year/1–2 years/3–4 years/5 years/more than 5 years*.

### 2.4 Statistical Methods

The prevalence and distribution of the variables (gender, age, MDAS) were presented as means and standard deviations (SD). The distribution of participants by background and oral health, oral health‐related behaviors, and drug use variables were presented in terms of frequencies and proportions. The significance of the association between categorized MDAS and the background factors, self‐reported oral health and oral health‐related behaviors, dental attendance and self‐reported etiology, and previous dental treatment and dental fear was analyzed using Pearson's *χ* ^2^ and Fisher's Exact test. ANOVA was used for continuous variables. Statistical significance was set to *p* < 0.05. Logistic regression models were used to estimate odds ratios (OR) and 95% confidence intervals (CI). Recorded data were analyzed using SPSS software version 28.0 Inc., Chicago, Ill., USA.

For analysis, the variables were dichotomized as follows: age (< 35 and ≥ 35 years), education level (compulsory/second or third degree), marital status (single/married or co‐habiting), work–life (employed/not employed), smoking and the use of snus (active/not smoking), and use of alcohol (no or monthly/weekly or more often). The main illicit drugs among the illicit drug users were grouped as follows: amphetamine, opioids, amphetamine and opioids, and cannabis. Mental disorders were categorized as follows: no/yes, depression (yes), general anxiety/panic attack (yes), psychosis/schizophrenia (yes), and personality disorder (yes). The following prescribed drugs were considered: antidepressants (Anatomical Therapeutic Chemical (ATC)‐code N06A), antiepileptics (N03A), antipsychotics (N05A), anxiolytics (N05B and N05C), and insomnia drugs (N05CF and N05CH).

### 2.5 Ethical Approval

The regional medical research ethics committee of the Wellbeing Services County of Northern Ostrobothnia gave permission to conduct the research (September 2020, EETTMK: 85/2020). Research approval was also obtained from the City of Oulu (June 2020, §45/2020 and February 2021, §12/2021) and from the “Friends of the Youth” Registered Association (August 2020).

## 3 Results

The study population was predominantly male (66%), and two‐thirds were single. The mean age was 33 years (SD 8.0, minimum 20 years and maximum 65 years). As reported by the participants, 71% had a medical condition needing medication and 45% had a mental health disorder. The average duration of drug use in the study population was 12.8 years (SD 5.94, a minimum of 1 year and a maximum of 26 years). The most commonly used drugs were opioids and amphetamine. Nearly half of the study population had completed only the compulsory education of 9 years and the other half had secondary or even tertiary education. The majority were either unemployed, on disability pension, or students, while only one in 10 was employed. The majority also smoked, two out of five used snus, and one‐fourth used alcohol weekly or more frequently (Table 1).

**Table 1:** Distribution of participants considering dental fear in association with background factors.

Variables, *n* (%)	MDAS, *n* (%)			Total, *n* (%)	*p*‐value
	MDAS < 10	MDAS 10–18	MDAS ≥ 19		
Illicit drug users	31 (26.5)	58 (49.6)	28 (23.9)	117 (100.0)	
Gender					
Women	9 (22.5)	17 (42.5)	14 (35.0)	40 (34.2)	0.137
Men	22 (28.6)	41 (53.2)	14 (18.2)	77 (65.8)	
Age					
< 35	19 (24.1)	39 (49.4)	21 (26.6)	79 (67.5)	0.538
≥ 35	12 (31.6)	19 (50.0)	7 (18.4)	38 (32.5)	
Education					
Compulsory	12 (22.2)	27 (50.0)	15 (27.8)	54 (46.2)	0.528
Second and third degree	19 (30.2)	31 (49.2)	13 (20.6)	63 (53.8)	
Marital status					
Single	24 (30.4)	35 (44.3)	20 (25.3)	79 (67.5)	0.242
Married or co‐habiting	7 (18.4)	23 (60.5)	8 (21.1)	38 (32.5)	
Work–life					
Employed	5 (41.7)	5 (41.7)	2 (16.7)	12 (10.3)	0.522^a^
Not employed^b^	26 (24.8)	53 (50.5)	26 (24.8)	105 (89.7)	
Smoking					
Active	26 (27.1)	46 (47.9)	24 (25.0)	96 (82.1)	0.779
Not smoking^c^	5 (23.8)	12 (57.1)	4 (19.0)	21 (17.9)	
Snus					
Active	7 (16.3)	27 (62.8)	9 (20.9)	43 (36.8)	0.073
Not using^c^	24 (32.4)	31 (41.9)	19 (25.7)	74 (63.2)	
Alcohol					
No or monthly	25 (28.4)	40 (45.5)	23 (26.1)	88 (75.2)	0.312
Weekly or more	6 (20.7)	18 (62.1)	5 (17.2)	29 (24.8)	
Main illicit drug (*n* = 116)^d^					
Cannabis	3 (30.0)	4 (40.9)	3 (30.0)	10 (8.6)	0.577^a^
Amphetamine	9 (32.1)	14 (50.0)	5 (17.9)	28 (24.1)	
Opioids	16 (25.8)	33 (53.2)	13 (20.9)	62 (53.5)	
Amphetamine + opioids	3 (18.8)	6 (37.5)	7 (43.8)	16 (13.8)	
In drug rehab					
Yes	6 (15.4)	18 (46.2)	15 (38.5)	39 (33.3)	0.018
No	25 (32.1)	40 (51.3)	13 (16.7)	78 (66.7)	
Prescribed drugs					
Antidepressants	7 (22.6)	11 (35.5)	13 (41.9)	31 (26.5)	0.022
Antipsychotics	9 (33.3)	11 (40.7)	7 (25.9)	27 (23.1)	0.554
Antiepileptics	6 (28.6)	10 (47.6)	5 (23.8)	21 (17.9)	1.000
Anxiolytics	4 (26.7)	8 (53.3)	3 (20.0)	15 (12.8)	0.939
Insomnia drugs	4 (33.3)	4 (33.3)	4 (33.3)	12 (10.3)	0.484
Chronic diseases requiring medication					
Yes	23 (27.4)	40 (47.6)	21 (25.0)	84 (71.8)	0.773
No	8 (24.2)	18 (54.5)	7 (21.2)	33 (28.2)	
Mental disorder					
Yes	17 (32.1)	23 (43.4)	13 (24.5)	53 (45.3)	0.400
No	14 (21.9)	35 (54.7)	15 (23.4)	64 (54.7)	
Depression	7 (25.9)	11 (40.7)	9 (33.3)	27 (23.1)	0.428
General anxiety/panic attack	6 (26.1)	14 (60.9)	3 (13.0)	23 (19.7)	0.367
Psychosis/Schizophrenia	6 (66.7)	3 (33.3)	0	9 (7.70)	0.014^a^
Personality disorder	1 (20.0)	3 (60.0)	1 (20.0)	5 (4.30)	1.000^a^

^a^ Fisher's exact test.

b Unemployed or retired or student.

c Never or former user.

^d^ One participant missing.

According to the single question, two‐thirds reported having dental fear. According to the MDAS, three‐fourths had at least moderate dental fear (MDAS ≥ 10), and of those, a third had severe dental fear (Table 1). The average MDAS sum score showed moderate dental fear [14.0 (SD 5.7)]. One in three was in drug rehabilitation, and they reported significantly more dental fear than the rest of the participants (*p* < 0.05).

Almost three out of four reported having a chronic disease that required medication and nearly a half reported mental disorders. The most common mental disorders were depression (23.1%) and general anxiety or panic attacks (19.7%) (Table 1). More than one‐fourth of the participants reported using antidepressants (26.5%) and more than one‐fifth used antipsychotics (23.1%). Those using antidepressants reported significantly more dental fear than the rest, whereas those with psychosis or schizophrenia reported significantly less dental fear than the rest (Table 1).

Self‐reported poor oral health as well as the dental treatment need were associated with severe dental fear. Those with severe dental fear frequently reported pain and teeth needing extractions. Nine in 10 had received fillings, which was significantly associated with dental fear (*p* = 0.036). In addition, self‐reported teeth needing extraction was also associated with dental fear, but not statistically significant (*p* = 0.056). Only about 10% attended dental care regularly, while almost 15% never did (Table 2).

**Table 2:** Distribution of participants considering self‐reported oral health and dental attendance in association with dental fear, *n* = 117.

	MDAS, *n* (%)			Total, *n* (%) 117	
Variables, *n* (%)	MDAS < 10 31 (26.5)	MDAS 10–18 58 (49.6)	MDAS ≥ 19 28 (23.9)		*p*‐value
Dental condition					
Poor or rather poor	15 (26.8)	26 (46.4)	15 (26.8)	56 (47.9)	0.941
Average	9 (26.5)	17 (50.0)	8 (23.5)	34 (29.1)	
Fairly good or good	7 (25.9)	15 (55.6)	5 (18.5)	27 (23.0)	
Oral health (yes)					
Cavities	24 (26.4)	45 (49.5)	22 (24.2)	91 (77.8)	1.000
Bleeding of gums while brushing	14 (23.7)	27 (45.8)	18 (30.5)	59 (50.4)	0.270
Tooth or teeth needing extractions	8 (15.4)	30 (57.7)	14 (26.9)	52 (44.4)	0.056
Pain or other symptoms	13 (28.9)	19 (42.2)	13 (28.9)	45 (38.5)	0.438
Healthy dentition	2 (9.5)	15 (71.4)	4 (19.0)	21 (17.9)	0.068
Dental treatment need					
Yes	30 (27.0)	54 (48.6)	27 (24.3)	111 (94.9)	0.872^a^
No	1 (16.7)	4 (66.7)	1 (16.7)	6 (5.1)	
Previous dental treatment (yes)					
Fillings	25 (23.4)	56 (52.3)	26 (24.3)	107 (91.5)	0.038^a^
Scaling	25 (26.0)	48 (50.0)	23 (24.0)	96 (82.1)	1.000
Individual dietary counseling	10 (22.2)	25 (55.6)	10 (22.2)	45 (38.5)	0.564
Individual oral hygiene counseling	25 (26.0)	46 (47.9)	25 (26.0)	96 (82.1)	0.572
Orthodontics	14 (23.3)	32 (53.3)	14 (23.3)	60 (51.3)	0.674
Extractions	25 (25.5)	47 (48.0)	26 (26.5)	98 (83.8)	0.348
Dental attendance					
Never	6 (37.5)	5 (31.3)	5 (31.3)	16 (13.7)	0.572^a^
If necessary due to pain	21 (24.1)	46 (52.9)	20 (23.0)	87 (74.3)	
Regularly for dental examination	4 (28.6)	7 (50.0)	3 (21.4)	14 (12.0)	
Previous dental visit					
More than 5 years or do not recall	7 (30.4)	8 (34.8)	8 (34.8)	23 (19.7)	0.534
3–5 years ago	5 (29.4)	10 (58.8)	2 (11.8)	17 (14.5)	
1–2 years	5 (17.4)	15 (53.6)	8 (28.6)	28 (23.9)	
< 1 year	14 (28.6)	25 (51.0)	10 (20.4)	49 (41.9)	
Previous dental visit due to pain (*n* = 116)					
Yes	20 (24.1)	40 (48.2)	23 (27.7)	83 (71.6)	0.198
No	11 (33.3)	18 (54.5)	4 (12.1)	33 (28.4)	
Satisfaction due to dental appearance (*n* = 115)					
Extremely not satisfied	4 (21.1)	7 (36.8)	8 (42.1)	19 (16.2)	0.352^a^
Not or rather not satisfied	11 (29.7)	21 (56.8)	5 (13.5)	37 (31.6)	
Satisfied or rather satisfied	13 (25.5)	26 (51.0)	12 (23.5)	51 (43.6)	
Extremely satisfied	1 (12.5)	4 (50.0)	3 (37.5)	8 (6.8)	

^a^ Fisher's exact test.

Men more frequently reported painful or difficult dental experiences (*p* = 0.041), poor dental experiences of family members or friends, excessive dental treatment needs, and health problems and mental health disorders as causes of their dental fear. Poor behavior by dental personnel was reported more often by women as the cause of their dental fear and they also had a higher total number of etiological factors for dental fear than men. Men used sedation in dental care more often than women (Table 3). Dental fear was significantly associated with painful or difficult dental experiences (*p* = 0.001) and poor behavior by dental personnel (*p* = 0.001), health problems and mental health disorders (*p* = 0.033), and the total number of etiological factors (*p* =0.001). About 28% of those with moderate and 40% of those with severe dental fear had received oral conscious sedation, compared with about 10% of those with mild dental fear (*p* = 00.28), whereas only eight participants had received either nitrous oxide in dental care or dental general anesthesia (DGA) (Table 3).

**Table 3:** Distribution of participants concerning self‐reported etiology and treatment of dental fear.

Variables, *n* (%)	MDAS, *n* (%)				Gender		Total, *n* (%)
	< 10 31 (%)	10–18 58 (%)	≥ 19 28 (%)	Total	Women	Men	
Causes for dental fear (*n* = 117–8109)							
Painful or difficult experiences^a^	2 (3.1)	37 (56.9)	26 (40.0)	65 (55.6) *p*: 0.001	27 (41.5)	38 (58.5)	65 (55.6) *p*: 0.041
Poor dental experiences of family or friends^a^	2 (10.5)	10 (52.6)	7 (36.8)	19 (17.4) *p*: 0.231^c^	7 (36.8)	12 (63.2)	19 (17.4)
Excessive dental treatment need^a^	3 (10.7)	15 (53.6)	10 (35.7)	28 (25.7) *p*: 0.100	6 (21.6)	22 (78.6)	28 (25.7)
General health and psychic problems^a^	3 (10.0)	15 (50.0)	12 (40.0)	30 (27.5) *p*: 0.033	11 (36.7)	19 (63.3)	30 (27.5) *p*: 0.821
Poor behavior of dental personnel^a^	0	9 (47.4)	10 (52.6)	19 (17.4) *p*: 0.001^c^	10 (52.6)	9 (47.4)	19 (17.4)
How many causes of dental fear do you have? mean (SD)	0.37 (0.839)	1.56 (1.259)	2.32 (1.188)	1.46 (1.339)	1.65 (1.160)	1.37 (1.419)	
	*p*‐value 0.001^b^				*p*‐value 0.304^b^		
Due to dental fear	
Nitrogen oxide used							
Yes	0	2 (66.7)	1 (33.3)	3 (2.6)	0	3 (100.0)	3 (2.6)
No	31 (27.2)	56 (49.1)	27 (23.7)	114 (97.4)	40 (34.8)	75 (62.5)	115 (98.3)
*p*‐value	*p*: 0.609^c^				*p*: 0.550^c^		
Oral conscious sedation							
Yes	3 (10.0)	16 (53.3)	11 (36.7)	30 (25.6)	8 (25.8)	23 (74.2)	31 (26.5)
No	28 (32.2)	42 (48.3)	17 (19.5)	87 (74.4)	32 (36.8)	55 (63.2)	87 (74.4)
*p*‐value	*p*: 0.028				*p*: 0282		
DGA							
Yes	0	3 (60.0)	2 (40.0)	5 (4.3)	0	5 (100.0)	5 (4.3)
No	31 (27.7)	55 (49.1)	26 (23.2)	112 (95.7)	40 (35.4)	73 (64.6)	113 (96.6)
*p*‐value	*p*: 0.361^c^				*p*: 0651^c^		

a Eight participants missing.

^b^ ANOVA test.

^c^ Fisher's exact test.

The unadjusted regression model showed statistically significant associations between severe dental fear, attending drug rehabilitation, and taking antidepressants. Male gender was a protective factor (Table 4). After adjusting for several confounding factors (age, gender, education, marital status, prescribed antidepressants, and presence of psychosis/schizophrenia) an association remained between at least moderate dental fear and previous painful or difficult dental experiences, poor behavior by dental personnel, multiple causes of dental fear, history of taking oral conscious sedation, and previous tooth extractions and fillings (OR 8.1, CI: 1.8–36.0.) (Tables 4 and 5). A significant association with severe dental fear was found before and after adjusting for variables. This association was linked to painful or difficult dental experiences, poor behavior by dental personnel, and multiple causes of dental fear (Tables 4 and 5).

**Table 4:** Unadjusted association between background factors and MDAS.

Variables	MDAS OR (95% CI)	
	≥ 10	≥ 19
Gender		
Women	1.0	1.0
Men	0.7 (0.3–1.8)	0.4 (0.2–1.0)*
Age		
< 35	1.0	1.0
≥ 35	0.7 (0.3–1.6)	0.6 (0.2–1.6)
Education		
Compulsory	1.0	1.0
Second and third degree	0.7 (0.3–1.5)	0.7 (0.3–1.6)
In drug rehab		
No	1.0	1.0
Yes	2.6 (1.0–7.0)	3.1 (1.3–7.5)*
Mental disorder		
No	1.0	1.0
Yes	0.6 (0.3–1.3)	1.1 (0.4–2.6)
Depression	1.0 (0.4–2.8)	1.9 (0.7–4.8)
General anxiety/panic attack	1.0 (0.4–2.9)	0.4 (0.1–1.5)
Psychosis	0.2 (0.0–0.7)*	0
Personality disorder	1.5 (0.2–13.6)	0.8 (0.1–7.4)
Prescribed drugs		
Antidepressants	1.3 (0.5–3.5)	3.4 (1.4–8.5)*
Antiepileptics	0.9 (0.3–2.5)	1.0 (0.3–3.0)
Antipsychotics	0.7 (0.3–1.7)	1.2 (0.4–3.1)
Anxiolytics	1.0 (0.3–3.4)	0.8 (0.2–3.0)
Insomnia drugs	0.7 (0.2–2.5)	1.7 (0.5–6.1)
Drug of choice^a^		
Cannabis	1.0	1.0
Amphetamine	0.9 (0.2–4.3)	0.5 (0.0–2.7)
Opioids	1.2 (0.3–5.3)	0.6 (0.1–2.7)
Amphetamine + opioids	1.8 (0.3–11.8)	1.8 (0.3–9.7)
Causes for dental fear		
Painful or difficult experiences (yes)	39.4 (8.6–181.0)*	14.3 (3.2–64.4)*
Poor dental experiences of family or friends (yes)	3.2 (0.7–15.0)	1.9 (0.7–5.6)
Excessive dental treatment need (yes)	3.5 (1.0–12.5)	1.0 (0.8–5.0)
General health and psychic problems (yes)	3.9 (1.1–13.9)*	2.7 (1.1–6.7)*
Poor behavior of dental personnel (yes)	0	4.5 (1.6–12.7)*
How many causes of dental fear do you have?	4.2 (2.1–8.1)*	2.0 (1.4–2.8)*
Oral conscious sedation		
No	1.0	1.0
Yes	4.3 (1.2–15.3)*	2.4 (1.0–5.9)
Teeth needing extraction		
No	1.0	1.0
Yes	3.1 (1.2–7.5)*	1.3 (0.6–3.2)
Fillings done in the past		
No	1.0	1.0
Yes	4.9 (1.3–18.8)*	1.3 (0.3–6.4)

Abbreviations: CI, confidence interval; OR, odds ratio.

a One participant missing.

* Statistically significant at *p* < 0.05.

**Table 5:** Adjusted association between background factors and MDAS.

Variables	MDAS OR (95% CI)	
	≥ 10	≥ 19
In drug rehab		
No	1.0	1.0
Yes	2.3 (0.8–6.8)	2.3 (0.9–6.0)
Causes for dental fear		
Painful or difficult experiences (yes)	52.3 (9.3–303)*	10.8 (2.3–52.0)*
General health and psychic problems (yes)	3.3 (0.9–12.6)	2.5 (0.9–6.8)
Poor behavior of dental personnel (yes)	—	4.1 (1.2–13.9)^a^
How many causes of dental fear do you have?	5.0 (2.2–11.2)*	1.9 (1.3–2.8)*
Oral conscious sedation		
No	1.0	1.0
Yes	5.0 (1.3–20.1)*	3.1 (1.1–8.6)*
Teeth needing extraction		
No	1.0	1.0
Yes	3.8 (1.9–10.9)*	1.8 (0.7–4.9)
Fillings		
No	1.0	1.0
Yes	8.1 (1.8–35.9)*	1.9 (0.3–10.9)

*Note:* Adjusted for gender, age, education, marital status, prescribed antidepressants, and presence of psychosis/schizophrenia.

Abbreviations: CI, confidence interval; MDAS, modified dental anxiety scale; OR, odds ratio.

^a^ One participant missing.

* Statistically significant at *p* < 0.05.

## 4 Discussion

The study population in this cross‐sectional survey comprised present and former users of illicit drugs in their thirties. Half of them reported at least moderate dental fear, and almost one‐fourth severe dental fear; women reported severe fear more frequently than men. Dental fear was significantly associated with the use of antidepressants and being in drug rehabilitation, but especially with previous dental experiences: receiving fillings, painful or difficult dental experiences, and poor behavior by dental personnel, as well as multiple etiological causes of dental fear.

The reported dental fear among illicit drug users in the current study exceeds that of the general population, in which one‐third report having moderate and 10% severe dental fear (Pohjola et al. 2008; Liinavuori et al. 2016). Worldwide, the prevalence of severe fear has been reported as being approximately 15% (Silveira et al. 2021). In the current study, women reported more fear than men, which is in line with previous studies (Silveira et al. 2021), and male gender was found to be a protective factor for severe dental fear. Female gender, young age, low education level as well as single marital status have often been found to correlate positively with dental fear (Lahti et al. 2007; Pohjola et al. 2008, 2013; Svensson, Hakeberg, and Boman 2016; Armfield, Spencer, and Stewart 2006). These factors were also present in the current study population; participants were often single, unemployed, or on disability pension, and half of them had completed only compulsory schooling. Surprisingly, despite the high MDAS mean score (14), no statistically significant associations were discovered between the traditional etiological factors and dental fear scores. Only being in drug rehabilitation was significantly associated with dental fear; the reason for this can only be speculated.

According to the literature, alcohol consumption, smoking, and use of snus are also associated with dental fear (Pohjola et al. 2013). In the current study, almost everyone smoked, about a third used snus, and one‐fourth consumed alcohol frequently. Nevertheless, these behaviors were not associated with dental fear. All of this suggests that traditional associations with dental fear are not observed among illicit drug users.

The poor oral health of illicit drug users has been explained by unhealthy behaviors and chaotic lifestyles as well as neglected self‐care (Robinson, Acquah, and Gibson 2005). The poor self‐reported oral health discovered in the current study is in line with several previous studies on the oral health of illicit drug users (Baghaie et al. 2017; Teoh, Moses, and McCullough 2019; Rossow 2021, Yazdanian et al. 2020; Cossa et al. 2020). Poor oral health inevitably leads to pain, which is a key element in the vicious circle of dental fear and irregular dental attendance (Armfield 2013, Kämppi et al. 2022). In the current study, only 10% of the participants reported attending dental care regularly, and a somewhat bigger proportion never and the rest if needed. Illicit drug use combined with dental fear may have led to irregular, pain‐driven dental attendance. However, no significant association between dental fear and attendance was discovered.

The participants here reported having undergone various dental procedures in the past, with half even receiving orthodontic care. Having fillings was significantly associated with dental fear. Indeed, dental caries still exists, practically all had enamel caries lesions. The strong association between dental fear and painful experiences emphasizes the role of pediatric and general dentistry in making all possible efforts to prevent both dental caries and fear especially among those with challenges in life. Here, too, poor behavior by dental personnel was associated with dental fear. Kankaala, Kaakinen, and Anttonen (2022) showed that people with at least a moderate level of dental fear reported dental personnel and dental treatments as key elements for improving dental care.

The etiology of dental fear is known to be complex (Beaton, Freeman, and Humphris 2014; Armfield 2008). Dental fear can partially be explained by its endogenous etiology (Weiner and Sheehan 1990), which means that some people are prone to psychological and anxiety disorders, manifested in generalized anxiety, behavioral problems, and a variety of severe fears as well as social phobias and depression (Armfield 2006, 2008; Locker, Poulton, and Thomson 2001; Stenebrand, Wide Boman, and Hakeberg 2013; Pohjola et al. 2011; Halonen et al. 2018). Illicit drug users often have psychological disorders and multiple chronic diseases (Sihvola et al. 2008; Chen and Lin 2009; Brienza et al. 2000), which was also the case in the current study. Having a psychosis diagnosis was associated with moderate dental fear, while being prescribed antidepressant medication was associated with severe dental fear. This study shows that addictive behaviors, physical and mental disorders, as well as dental fear, seem to build up in some individuals. The reasons and mechanisms for this are beyond the scope of this work and require further investigation.

After adjusting the data for marital status, age, gender, educational status, use of antidepressants, and present psychosis and schizophrenia, an association remained between oral health‐related factors and at least moderate dental fear. The finding emphasizes the importance of good oral health, as well as positive experiences in a dental office throughout childhood and adolescence and into adulthood for everyone.

Even if conscious sedation or DGA does not eliminate dental fear (Kankaala et al. 2019), they are commonly used to enable dental work. However, psychological and cognitive behavioral therapy (CBT) and behavioral therapy (BT) are the key instruments for a dentist to help their patients cope with their dental fear (Wide Boman et al. 2013, Kankaala et al. 2019). In the present study, oral conscious sedation was the most frequently used sedation method specifically for those with severe and moderate dental fear. Nitrous oxide and DGA were seldom used. DGA is the last resort for treating fearful patients, but among drug users, this type of treatment is justified because poor oral health is an essential systemic risk. Besides, the users of illicit drugs may not be able to cope with long procedures, so the use of conscious sedation is indicated; nevertheless, psychological methods can be used for them, for example, to improve their dental attendance (Kvale, Berggren, and Milgrom 2004).

There is limited literature on dental fear among illicit drug users, which adds to the value of this study. In addition, the present study is comprehensive and versatile. The participants comprised both active and former illicit drug users, individuals undergoing pharmaceutical substitution and maintenance therapy, or medically assisted drug rehabilitation. Altogether, eight different substance abuse service units in the Oulu region were involved in the study. The examinations and interviews were carried out by trained and calibrated dentists in a familiar and safe environment for the participants. This arrangement enabled data collection, as the threshold for making the effort to come to the dental clinic would have most likely been too high for the participants. To get responses, a research‐assisted, face‐to‐face interview was also used in carrying out the survey. Validated questionnaires were used to assess dental fear and its associated factors. The use of these is also a strength of this study.

A limitation of this study is the lack of psychiatric and somatic diagnoses as well as medications; this information was self‐reported by the participants. The reliability of this information is open to speculation. The number of participants was limited and less than estimated in power calculations due to the challenges in the field study among this study population, and the long data collection period which was caused by the COVID‐19 pandemic. There are no controls in this study, but dental fear among Finnish adults has been widely studied (Pohjola et al. 2008; Liinavuori et al. 2016, 2019; Vainionpää et al. 2019) which allows for comparisons.

## 5 Conclusions

Users of illicit drugs are often neglected in dental care, as they mostly seek only acute dental care. They often have psychic and physical conditions as well as dental fear, which are all linked to each other and may complicate dental treatments. An association was found between dental fear and previous painful experiences and poor behavior by dental personnel. The abovementioned factors and poor oral health highlight both the role of prevention for this patient risk group of patients, as well as the need to practice their communication skills in dental care.

## Author Contributions

Raija Vainionpää and Antti Tiisanoja were responsible for the design of the study and planning and execution of the field phase. Raija Vainionpää was responsible for getting permission, and she participated in the analyses and preparation of the manuscript. Antti Tiisanoja was responsible for the statistics, analyses, and preparation of the manuscript. Outi Kokkola participated in the field phase and preparation of the manuscript. Pirkko Riipinen gave her expertise in the field of psychiatry in the analyses and preparation of the manuscript. Vuokko Anttonen participated in the study design, permissions, planning of the field phase, analyses, and preparation of the manuscript.

## Conflicts of Interest

The authors declare no conflicts of interest.

## Data Availability

The authors have nothing to report.
